# Targeting Mechanics-Induced Fibroblast Activation through CD44-RhoA-YAP Pathway Ameliorates Crystalline Silica-Induced Silicosis

**DOI:** 10.7150/thno.35665

**Published:** 2019-07-09

**Authors:** Siyi Li, Chao Li, Yiting Zhang, Xiu He, Xi Chen, Xinning Zeng, Fangwei Liu, Ying Chen, Jie Chen

**Affiliations:** Division of Pneumoconiosis, School of Public Health, China Medical University, Shenyang, PR China.

**Keywords:** silicosis therapy, mechanics, fibroblast activation, Yes-associated protein

## Abstract

Silicosis is pneumoconiosis of the lung, usually resulting from prolonged exposure to crystalline silica (CS). The hallmark of silicosis is excessive extracellular matrix (ECM) deposition produced by activated fibroblasts. Recent work demonstrated that excessive ECM-forming mechanical cues play an essential role in promoting fibroblast activation and perpetuating fibrotic pathologies. However, the detailed molecular mechanism still needs to be uncovered.

**Methods**: NIH-3T3 fibroblasts were cultured on either 1 kappa (soft) or 60 kappa (stiff) gel-coated coverslips. A series of knockdown and reverse experiments *in vitro* were performed to establish the signaling for mechanics-induced fibroblast activation. An experimental model of silicosis was established by one-time intratracheal instillation of CS suspension. The cluster of differentiation 44 (CD44) antibody (IM7), dihydrotanshinone I (DHI) and verteporfin (VP) were used to explore the effect of CD44-RhoA-YAP signaling blockade on mechanics-induced fibroblast activation and CS-induced pulmonary fibrosis.

**Results**: Matrix stiffness could induce nuclear translocation of the Yes-associated protein (YAP) through CD44 in fibroblasts. This effect required RhoA activity and F-actin cytoskeleton polymerization but was independent of Hippo pathway kinases, Mst 1 and Lats 1, forming CD44-RhoA-YAP signaling pathway. Pharmacological upstream blocking by CD44 antibody or downstream blockade of YAP by DHI or VP could attenuate fibroblast migration, invasion, proliferation, and collagen deposition. Furthermore, CD44-RhoA-YAP signaling blockade could alleviate CS-induced fibrosis and improve pulmonary function *in vivo*.

**Conclusion**: CD44-RhoA-YAP signaling mediates mechanics-induced fibroblast activation. Targeting this pathway could ameliorate crystalline silica-induced silicosis and provide a potential therapeutic strategy to mitigate fibrosis.

## Introduction

Occupational exposure to crystalline silica (CS) occurs in many industries and occupations. Long- term inhalation of CS leads to silicosis, which is characterized by chronic lung inflammation and progressive fibrosis 1. Persistent inflammation results in tissue damage, fibroblast proliferation and activation, and abnormal collagen deposition eventually leading to loss of lung functions and disability even death. Excessive extracellular matrix (ECM) deposition is the hallmark of silicosis. A major source of increased ECM is the dysregulated matrix synthesis by activated fibroblasts, which differentiate into myofibroblasts. At present, few effective therapies can reverse or even delay the progression of silicosis. Patients are usually provided supportive care with anti-inflammatory treatment to avoid complications. However, fibrogenesis-related research indicated that anti-inflammatory therapy alone has little effect on the progression of fibrogenesis even if the inflammation is well-controlled 2, 3. Thus, directly targeting fibroblasts or their differentiation into myofibroblasts may provide the clinical potential to mitigate fibrosis.

Fibroblasts can be activated by growth factor signaling, becoming α-smooth muscle actin (α-SMA)- positive cells 4. Recently, the extracellular matrix, with its most abundant component collagen, is emerging as a new crucial factor that influences cell behavior by imparting biochemical and mechanical cues. Tensional, compressive, and shear forces can be translated into biochemical signaling through a process known as mechano-transduction signaling 5. Mechanical cues can lead to fibroblast activation in multi-organs including lung, kidney, and liver 6-8. Yes-associated protein (YAP), the downstream effector of Hippo signaling cascade, known for influencing cell proliferation and apoptosis, can be regulated by matrix rigidity. Matrix stiffness-induced YAP activation contributes to fibroblast phenotypic conversion, increased collagen deposition, and cell proliferation by augmenting feed-forward cycles that enhance microenvironment stiffening. Although the cellular distribution of YAP has been reported to be positively regulated by matrix stiffness 9, 10, the underlying molecular mechanism by which YAP mechanically promotes the positive loop is still unclear and required to be further explored. Targeting the mechano-sensitivity or interrupting the cellular response may be a therapeutic approach with clinical potential.

Here, we investigated the upstream and downstream components of the YAP, making it a mechano-activation pathway and developed several strategies targeting the signaling cascade to block fibroblast activation. We demonstrated that cluster of differentiation 44 (CD44, a cell-surface glycoprotein) 11 might function as a sensor of matrix stiffness and play a crucial role in YAP-mediated fibroblast activation. A series of knockdown and reverse experiments *in vitro,* identified that the signaling transduction was dependent on RhoA activation and F-actin cytoskeleton polymerization, but not on Hippo pathway kinases Mst 1 and Lats 1. Furthermore, we utilized anti-CD44 antibody or gradient-dose dihydrotanshinone I (DHI, a lipophilic component of traditional Chinese medicine Salvia Miltiorrhiza Bunge) 12 as well as verteporfin (VP, a small molecule YAP inhibitor) 13 and verified that the CD44-RhoA-YAP signaling blockade could affect fibroblast function and postpone fibrosis progression in experimental silicosis. This thesis may shed light on a new molecular mechanism and a specific molecular target to postpone CS-induced fibrosis.

## Materials and Methods

### Animals and treatments

Male C57BL/6 mice (6-8 weeks) were purchased from SLAC Laboratory Animal Co. Ltd. (Shanghai, China). All mice were raised in a pathogen-free facility, provided with a standard mice feedstuff and water ad libitum. The animals were acclimatized for a week before starting the experiments. All animal experiments were approved by the Animal Care and Use Committee at China Medical University. The silicosis model mice were described previously 14-16.

Study 1: Male C57BL/6 mice received one-time intratracheal instillation of 50 μL CS suspension (10 mice per group). Mice were randomized to receive weekly intraperitoneal injection of rat anti-mouse CD44 antibody (IM7) or isotype control rat IgG2b (300 μg in 500 μL saline). As shown in Figure S1A, treatment in this study began at day 7 after CS instillation and all mice were sacrificed at indicated time points under anesthesia.

Study 2: Male C57BL/6 mice received one time either intratracheal instillation of 50 μL CS suspension or sterile saline (10 mice per group). Mice receiving CS suspension were randomized to receive I: DMSO vehicle control, II-IV: daily intragastrical administration of DHI at doses of 150, 75, or 37.5 mg/kg dissolved in DMSO, respectively, and V: intraperitoneal injection of verteporfin at the dose of 100 mg/kg dissolved in DMSO every other day. Treatments in this study began at day 7 after CS instillation and all mice were sacrificed at indicated times under anesthesia (Figure S1B-C). Lung tissues were obtained for further analyses.

### Pulmonary function assay

Body weight as well as minute volume, tidal volume, and breathing frequency of the male C57BL/6 mice, which received sterile saline, CS, CS+anti-CD44, CS+VP, and CS+DHI150 mg/kg were recorded prior to administering different treatments. These parameters were recorded again at week 1, 2, 3, 4, 5, 6, 7, and 8. We used minute volume, tidal volume, and breathing frequency relative to body weight to describe the mice pulmonary function.

### Cytotoxicity assay of DHI

The cytotoxicity of DHI was assessed by the MTT assay. NIH-3T3 fibroblasts cell line, purchased from the National Infrastructure of Cell Line Resource (Beijing, China) , were cultured in 96-well plates at an initial density of 5×10^4^ cells/well in a serum-free Dulbecco's Modified Eagle Medium (DMEM) overnight. Subsequently, NIH-3T3 fibroblasts were treated with DHI at different concentrations for 24 hours. Then DMEM was removed and replaced with 20 µL MTT (0.5 mg/mL) for 4 hours followed by 150 μL DMSO for 10 minutes. The absorbance of the dissolved formazan crystals was measured at 570 nm by a 96-well multimode plate reader (Figure S2).

### 2D cell culture and treatments

The 2D Col-gel was acquired from Bioruo (Beijing, China). NIH-3T3 fibroblasts were cultured at 37°C with 5% CO_2_ and grown in DMEM supplemented with 10% FBS, 100 U/mL penicillin, 100 μg/mL streptomycin, and 10 mM HEPES. Following overnight serum deprivation (DMEM without FBS), NIH-3T3 cells were seeded on coverslips or plastic dishes coated with either 1 kappa (soft) or 60 kappa (stiff) polyacrylamide hydrogels with sterile collagen to create a uniform, thin layer of ECM protein according to the manufacturer's protocol. Cells were treated with anti-CD44 antibody (IM7) 10μg/mL or isotype-matched control IgG antibody for 12 hours to block the upstream signaling pathway. Cells were treated with VP (250 nM) or DHI (37.5, 75 or 150 nM) for 12 hours for blocking the downstream signaling pathway.

### Transfection

Cells were seeded on soft (1 kappa) or stiff (60 kappa) gel-coated coverslips or plastic dishes using DMEM medium with 1% FBS and without antibiotics overnight before transfection with I: targeting siRNAs or equivalent amount of non-targeting siRNA, II: constitutively active RhoA (Q63L) plasmid or pcDNA3.1 for 48 hours. The siRNAs and plasmid were transfected into cells using LipoFiter^TM^ (Hanbio) according to the manufacturer's instructions. Transfection efficiency of each siRNA target or plasmid was confirmed via Western blotting (Figure S3), and cells were used for subsequent experiments. The sequences of siRNAs were as follows:

si-NC: 5′-AAUUCUCCGAACGUGUCACGUUU-3′; si-CD44: 5′-CGUGGAGAAAAAUGGUCGC-3′; si-RhoA: 5′-GACAUGCUUGCUCAUAGUCTT-3′; si-Rac1: 5′-GAUAACUCACCACUGUCCATT-3′; si-Cdc42: 5′-GACUCCUUUCUUGCUUGUUTT-3′; si-YAP: 5′-GACAUCUUCUGGUCAGAGA-3′.

The siRNAs were designed and synthesized by Ribobio (Guangzhou, China). The plasmid was designed and synthesized by Hanbio (Shanghai, China).

### Total and nuclear-cytosol protein extraction and Western blot analysis

Total protein was extracted from lung tissues or cells by RIPA lysis buffer containing protease inhibitor (Beyotime, China). Nuclear and cytoplasmic proteins were extracted from fresh lung tissues by a Nuclear-Cytosol Extraction Kit (Applygen Technologies Inc., Beijing, China) according to the manufacturer's instructions. For Western blot analysis, primary antibodies are specified in Table S1.

### Immunofluorescence

The treated cells were fixed with 4% paraformaldehyde for 15 minutes followed by permeabilization with 0.2% Triton X-100 for 10 minutes and blocked with 5% BSA for 30 minutes at room temperature. To visualize actin filaments, cells were incubated with FITC-conjugated phalloidine (Cytoskeleton, 70nM) for 30 minutes at room temperature. To visualize CD44 and YAP, cells were incubated with primary antibodies overnight at 4 °C. For immunofluorescence analysis, primary antibodies are specified in Table S1. Subsequently, the cells were incubated with Alexa Fluor 546-conjugated secondary antibody (1:200) for 1 hour at room temperature and stained with 4',6'- diamidino-2-phenylindole (DAPI) for 10 minutes to visualize nuclei. Fluorescent images were captured with an Olympus confocal microscope. YAP nuclear staining was quantified by counting the YAP staining-positive cells and then normalizing to the total cell number in each image. Three different fields were selected from a cell-cultured coverslip, and three parallel experiments for each treatment were performed. All data were evaluated to obtain a mean value for subsequent statistical analysis.

### Wound healing assay

Cells were seeded on stiff (60 kappa) gel-coated plastic dishes and cultured until the cells reached 70%-80% confluence. The monolayers were wounded by scratching with a sterile 20 μL pipette tip to create a straight linear scratch and washed with PBS to remove the detached cells followed by the previously described procedure. Wound gap was captured with a microscope and quantitatively evaluated with the Image J software.

### Transwell migration assay

1.5×10^4^ cells suspended in 200 μL of serum-free DMEM were seeded into the upper chamber of a Transwell insert coated with stiff (60 kappa) Col-gel, and 500 μL of serum-free DMEM was placed in the lower chamber overnight. Then, cells cultured in the upper chamber received different treatments, and the medium in the lower chamber was replaced with fresh DMEM containing 10% FBS. Cells remaining on the upper chamber were scraped off using a cotton swab, while cells traversed to reverse face of the membrane were placed in 0.5 mg/mL MTT for 4 hours followed by 500 μL DMSO for 10 minutes. The absorbance of the dissolved formazan crystals was measured at 570 nm by a 96-well multimode plate reader.

### Cell proliferation assay

The Cell Counting Kit-8 (MedChemExpress, China) was used to detect cell proliferation according to the manufacturer's instructions. Briefly, 5×10^4^ cells suspended in 100 μL of serum-free DMEM were seeded into 96-well plates coated with stiff (60 kappa) Col-gel overnight. Then, the cells received different treatments followed by 10 μL CCK-8 for 4 hours. Absorbance was measured at 490 nm by a 96-well multimode plate reader.

### Apoptosis assay

To detect cell apoptosis, Annexin V-PE and PI (BD Pharmingen, USA) staining were performed according to the manufacturer's instructions. Briefly, treated cells were incubated with Annexin V-FITC (5 μL) and Annexin V-PI (5 μL) for 10 minutes at room temperature in the dark. Expression of Annexin V and PI was detected by a flow cytometer (FACS). The Annexin V+/PI- represents early apoptotic cells and the Annexin V+/PI + represents late apoptotic cells.

### Luciferase reporter assay

1×10^5^ cells suspended in 100 μL of serum-free DMEM were seeded into 96-well plates coated with stiff (60 kappa) Col-gel overnight. Subsequently, cells were transfected with a Smad4-binding element luciferase reporter containing four repetitions of the GTCTAGAC using LipoFiter^TM^ (Hanbio) according to the manufacturer's instructions. Cell lysates were obtained, and luciferase activity was measured using a luciferase assay kit (Promega, USA).

### Quantitative PCR analysis

Total RNA was extracted from treated cells using Trizol (Life Technologies, USA) according to the manufacturer's protocol, and reverse transcribed into cDNA with the PrimeScript RT kit (Takara, China). Equivalent amounts of each cDNA sample were added to a SYBR Green Master Mix Kit (Takara, China). PCR primers used in this study are described in Table S2. The mRNA levels of *Tgf-β1, Pai, Il-6, Il-8, Fibronectin, and Collagen-1a1* were determined using the 2 ^-ΔΔCT^ method.

### ELISA assay

A tracheostomy was performed to obtain bronchoalveolar lavage fluid (BALF) by perfusing with 1ml sterile saline twice. The BALF was centrifuged at 1500 rpm for 8 minutes at 4°C. Levels of TNFα, IL-6, and IL-1β were measured by ELISA kits (R&D Systems, USA) according to the manufacturer's instructions. Absorbance was measured at 450 nm and 570 nm by a 96-well multimode plate reader. Concentrations are expressed as pg/mL.

### Hydroxyproline assay

To quantify lung collagen contents, the hydroxyproline (HYP) contents of the lungs were measured with a HYP kit (Nanjing Jian Cheng Institute, China) according to the manufacturer's instruction. The HYP contents were expressed as micrograms per gram (μg/g) of lung wet weight.

### Histological analysis and immunochemistry

Lung samples were fixed in 4% paraformaldehyde, embedded in paraffin and then sectioned (5 μm) to expose the main intrapulmonary bronchus. For histopathological analysis, hematoxylin and eosin (H&E) or Masson's trichrome staining were performed using a standard protocol to evaluate inflammation and fibrosis. For immunofluorescence and immunohistochemistry analysis, primary antibodies are specified in Table S1. The images were captured with a microscope.

### Statistical analysis

The data are presented as means ± SD. Differences between multiple groups were calculated using the one-way analysis of variance (ANOVA). The unpaired two-sided Student's t-test was used to calculate differences in measured variables. P<0.05 was considered to indicate a statistically significant result. Statistical analysis was performed using Origin Pro 8.0 or SPSS 19.0.

## Results

### Mechanics induces YAP nuclear localization and CD44 expression in fibroblasts

**Figure 1:**
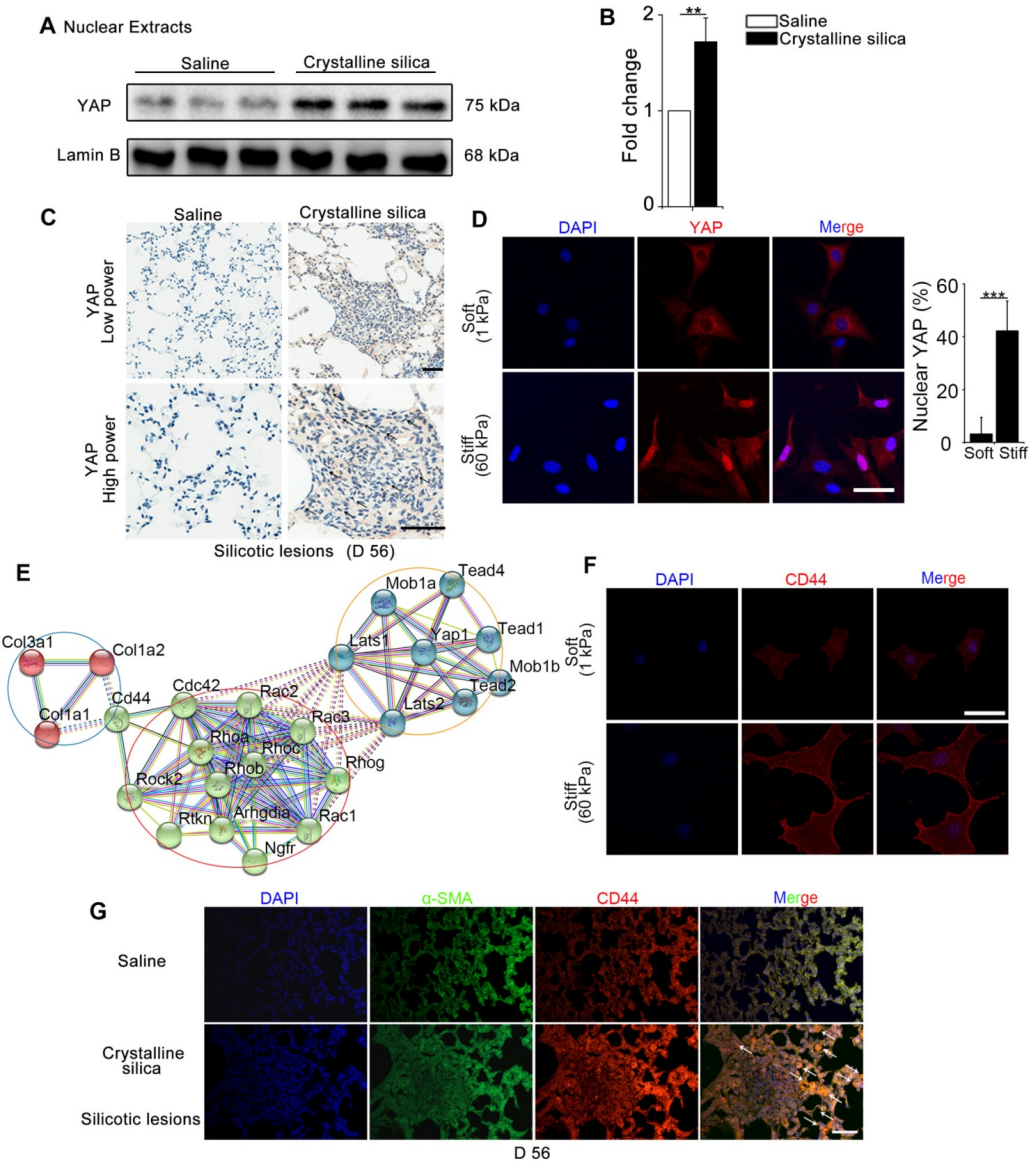
** Mechanics-induced YAP localization and CD44 expression in fibroblasts.** (**A-B**) Western blot analysis of YAP in nuclear extracts of lung tissues after different treatments at day 56. Lamin B was used as a loading control. Data shown are representative of three independent experiments. Error bars indicate mean ± SD (**, P < 0.01). (**C**) Distribution of YAP was determined by immunohistochemical staining in the lungs after saline or crystalline silica treatment at day 56. Arrows point to cells in the lung with silicotic lesions with predominantly nuclear YAP staining. Scale bar, 50 μm. (**D, F**) NIH-3T3 fibroblasts cultured on soft (1 kappa) or stiff (60 kappa) gel-coated coverslips were immunostained with an antibody recognizing YAP (D) and CD44 (F). The percentage of cells with predominantly nuclear YAP staining was quantified at D. Nuclei were counter-stained with DAPI. Scale bar, 50 μm (n=3; ***, P < 0.001). (**E**) Bioinformatic analysis of protein-protein interaction networks in STRING v.10. A screenshot from STRING shows a network associated with CD44 and YAP. The red nodes show extracellular matrix proteins; the green nodes show the association between CD44 and YAP; the blue nodes show cell signaling proteins in cytoplasm associated with YAP. (**G**) Immunofluorescent analysis of α-SMA and CD44 in lung sections with silicotic lesions after saline or crystalline silica treatment at day 56. Arrows point to fibroblasts expressing CD44. Representative images of the staining are shown (n=3). Scale bar, 100 μm.

YAP, as a major effector of Hippo signaling pathway, is reported to translocate into nucleus induced by mechanical force 9. The hallmark of silicosis is the increased stiffness of the lung. To explore the relationship between YAP and fibrogenesis, we examined nuclear YAP expression in the lungs of silicosis model mice and their controls. As shown in Figure 1A-C, western blot analysis and immunohistochemistry staining confirmed upregulated expression and nuclear localization of YAP in the CS-injured lung tissues. Furthermore, YAP nuclear localization was more pronounced in spindle-shaped cells in silicotic lesions (Figure 1C) suggesting its crucial role in fibroblasts. To confirm whether matrix stiffness induced YAP nuclear localization in fibroblasts, we cultured NIH-3T3 fibroblasts with collagen-1-coated soft gel (1 kappa) and stiff gel (60 kappa) to mimic the normal and injured fibrotic lung tissues, respectively. YAP immunostaining showed dramatically increased nuclear localization in NIH-3T3 fibroblasts cultured on stiff (60 kappa) gel-coated coverslips compared with those cultured on coverslips coated with soft gel (1 kappa) (Figure 1D). These results indicated that matrix stiffness- induced YAP nuclear translocation in fibroblasts might play a role in the progression of silicosis.

We further investigated the underlying molecular mechanism for the nuclear localization of YAP. Bioinformatic analysis of protein-protein interactions in STRING v.10 provided a protein network associated with YAP and matrix proteins, which clearly showed that collagens might interact with CD44 activating cytoplasmic signaling protein cascade (Figure 1E). Relevant to this information, we detected an apparent increase in CD44 in NIH-3T3 fibroblasts cultured on stiff gel-coated coverslips compared with the soft gel-cultured counterparts (Figure 1F and Figure S4). Besides, double immunostaining indicated higher expression of CD44 in α-SMA-positive myofibroblasts in the CS-injured lung tissues with silicotic lesions than in saline-treated mice (Figure 1G). These data indicated that CD44- induced nuclear translocation of YAP in fibroblasts might be associated with silicotic fibrogenesis.

### Mechanics regulates YAP activity through CD44-mediated RhoA activation rather than Rac and Cdc42

**Figure 2:**
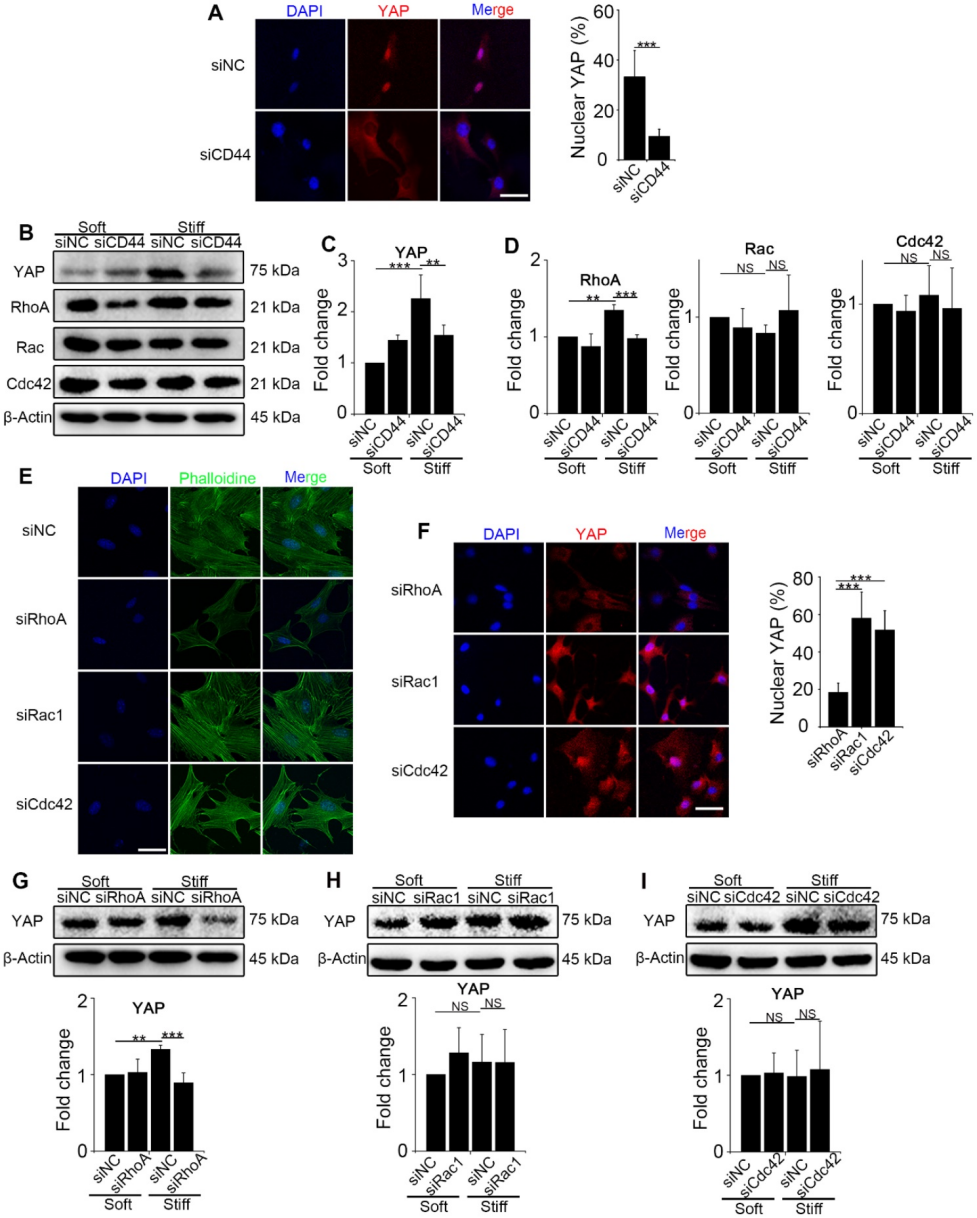
** CD44 acts through RhoA modulating YAP activation induced by matrix stiffness stimuli. (A-D**) NIH-3T3 fibroblasts were transfected with NC or CD44 siRNA on stiff (60 kappa) gel-coated coverslips for 48 hours. (**A**) NIH-3T3 fibroblasts were immunostained with an antibody recognizing YAP. The percentage of cells with predominantly nuclear YAP staining was quantified. Nuclei were counter-stained with DAPI (n=3; ***, P < 0.001). Scale bar, 50 μm. (**B-D**) Western blot analysis of YAP expression and Rho family proteins (RhoA, Rac and Cdc42). β-Actin was used as a loading control. Quantification of YAP (C) and Rho family proteins (D) is shown. (**E-F**) NIH-3T3 fibroblasts were transfected with NC or RhoA, Rac1, or Cdc42 siRNAs on stiff (60 kappa) gel-coated coverslips for 48 hours, immunostained with phalloidine to visualize F-actin (E), and an antibody recognizing YAP to visualize YAP localization (F) (n=3; ***, P < 0.001). The percentage of cells with predominantly nuclear YAP staining was quantified. Scale bar, 50 μm. (**G-I**) NIH-3T3 fibroblasts were transfected with NC or RhoA (G), Rac1 (H), and Cdc42 (I) siRNAs. Cell lysates were subjected to immunoblotting with YAP antibody. β-Actin was used as a loading control. Quantification of YAP level is shown. (**C-D and G-I**) Data shown are representative of three independent experiments. Error bars indicate mean ± SD (**, P < 0.01; ***, P < 0.001; NS, not significant).

To explore the interaction between CD44 and activated YAP, we first knocked down CD44 in NIH-3T3 fibroblasts with CD44 siRNA and cultured the cells on stiff (60 kappa) or soft (1 kappa) gel-coated coverslips. Immunostaining and Western blot analysis revealed that CD44 knockdown reduced YAP nuclear localization and expression in the fibroblasts cultured on the stiff matrix (Figure 2A-C). The bioinformatic analysis had also predicted that three major Rho family members RhoA, Rac, and Cdc42, may be involved in YAP activation. Hence, we hypothesized that CD44 mediates YAP activity through Rho family members. To test our hypothesis, we first performed Western blotting in the CD44 knockdown NIH-3T3 fibroblasts and found RhoA, rather than Rac or Cdc42 expression to be significantly reduced (Figure 2B and D). Next, we verified that CD44 regulated YAP activity through RhoA by transfecting NIH-3T3 fibroblasts with RhoA, Rac1, or Cdc42 siRNAs. As shown in Figure 2E, only RhoA knockdown could depolymerize the F-actin cytoskeleton (a major mechanical cue) of NIH-3T3 fibroblasts. Immunostaining and Western blot analysis showed that only RhoA knockdown could reduce YAP nuclear localization and expression (Figure 2F-G). By comparison, F-actin cytoskeleton and YAP activity were only marginally affected by Rac1 or Cdc42 knockdown (Figure 2E-F and H-I). These results suggested that RhoA is involved in CD44 modulation of YAP activation by matrix stiffness stimuli.

### RhoA regulates YAP activity through F-actin cytoskeleton polymerization/ depolymerization independent of Hippo pathway kinases

**Figure 3:**
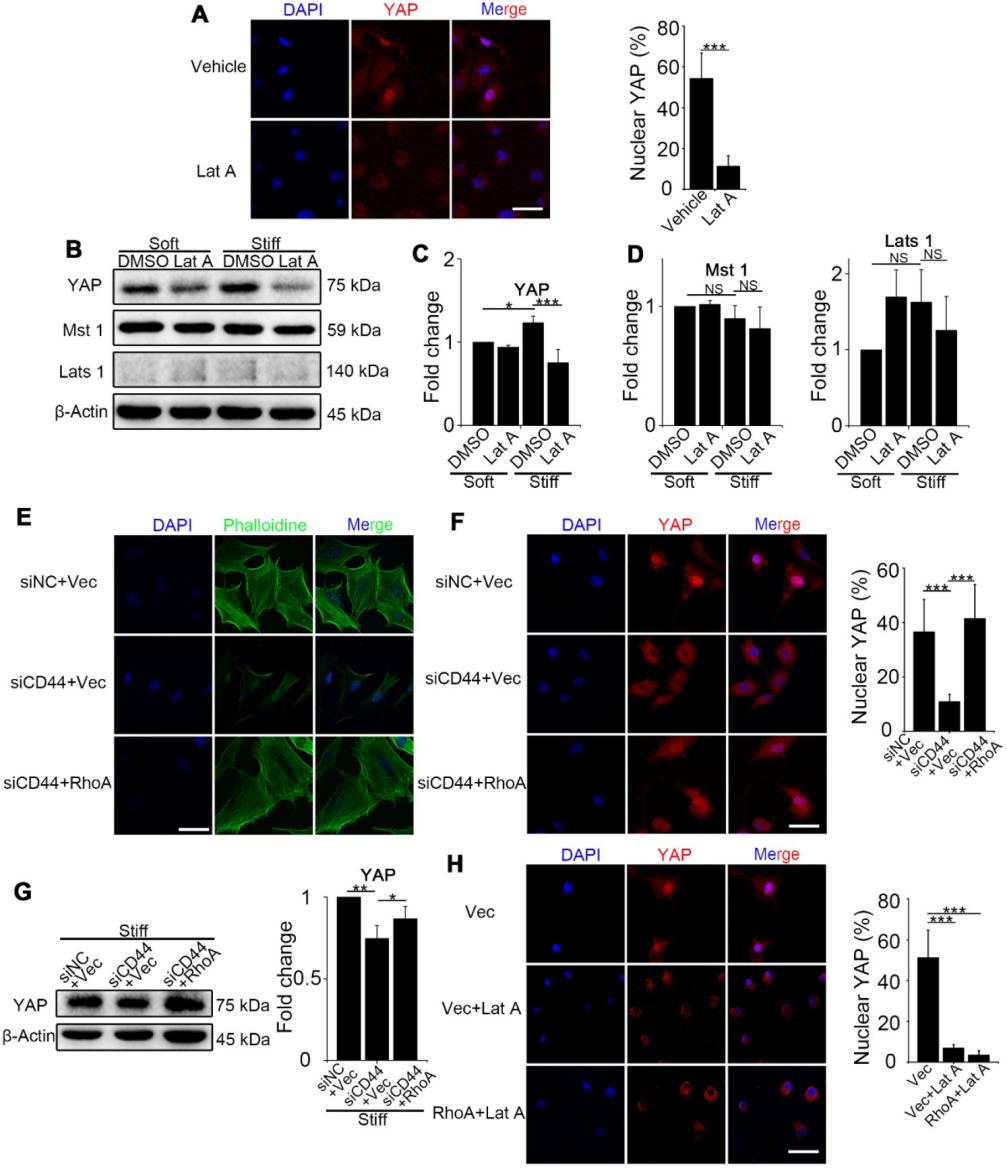
** RhoA acts through F-actin cytoskeleton to regulate YAP activation independent of the Hippo kinase cascade. (A-C**) NIH-3T3 fibroblasts grown on stiff (60 kappa) gel-coated coverslips were treated with or without 0.5 μM Lat A for 4 hours. (**A**) NIH-3T3 cells were immunostained with an antibody recognizing YAP. The percentage of cells with predominantly nuclear YAP staining was quantified. Nuclei were counter-stained with DAPI (n=3; ***, P < 0.001). Scale bar, 50 μm. (**B**) Western blot analysis of YAP and the Hippo kinase cascade proteins (Mst 1 and Lats 1) expression. β-Actin was used as a loading control. (**C-D**) Quantification of the indicated level is shown. (**E-G**) NIH-3T3 fibroblasts grown on stiff (60 kappa) gel-coated coverslips were co-transfected with NC/CD44 siRNA and vector/constitutively active RhoA-Q63L (ca-RhoA) plasmid for 48 hours. (**E, F**) NIH-3T3 cells were immunostained with phalloidine to visualize F-actin (E) and an antibody recognizing YAP to visualize YAP localization (F). (n=3; ***, P < 0.001). The percentage of cells with predominantly nuclear YAP staining was quantified. Scale bar, 50 μm. (**G**) Western blot analysis of YAP expression. β-Actin was used as a loading control. Quantification of YAP level is shown. (**H**) NIH-3T3 fibroblasts grown on stiff (60 kappa) gel-coated coverslips were transfected with vector/ca-RhoA plasmid for 48 hours and treated with or without Lat A for 4 hours, and immunostained with an antibody recognizing YAP. The percentage of cells with predominantly nuclear YAP staining was quantified. Nuclei were counter-stained with DAPI (n=3; ***, P < 0.001). Scale bar, 50 μm. (**C, D, G**) Data shown are representative of three independent experiments. Error bars indicate mean ± SD (*, P < 0.05; **, P < 0.01; ***, P < 0.001; NS, not significant).

We next investigated how RhoA regulates YAP activity. First, we examined NIH-3T3 fibroblasts treated with Lat A (F-actin inhibitor) followed by culturing on stiff gel-coated coverslips. We found that inhibiting F-actin cytoskeleton polymerization could downregulate YAP nuclear localization and expression (Figure 3A-C) by immunostaining and Western blot analysis. Of note, YAP alteration occurred in as short as 4 hours after Lat A treatment. To investigate whether F-actin influenced YAP activity via Hippo pathway kinases, we performed Western blotting of Mst 1 and Lats 1 and found that their expression did not change by Lat A treatment (Figure 3B and D). Collectively, the results suggested that Hippo pathway kinases may not be indispensable mediators in F-actin regulation of YAP activity. Thus, our results indicated that CD44 modulates RhoA activation, which, in turn, influences F-actin polymerization/depolymerization and may be a primary signaling pathway in matrix stiffness regulation of YAP activity.

To sort out the upstream and downstream relationship, we did a series of reverse experiments. First, we co-transfected CD44 siRNA and constitutively activated RhoA-Q63L (ca-RhoA) plasmid in fibroblasts. Results showed that ca-RhoA could reverse the CD44 knockdown effects on F-actin cytoskeleton depolymerization, YAP cytoplasmic retention, and decreased expression (Figure 3E-G). We also treated ca-RhoA plasmid-transfected fibroblasts with Lat A. Figure 3H showed that Lat A could reverse the effect of RhoA activation on YAP nuclear localization. Importantly, these results revealed that CD44-induced YAP activation acts in parallel with Hippo pathway kinases and requires RhoA activity for F-actin cytoskeleton polymerization.

### CD44-RhoA-YAP signaling pathway blockade affects fibroblast function imposed by matrix stiffness stimuli

**Figure 4:**
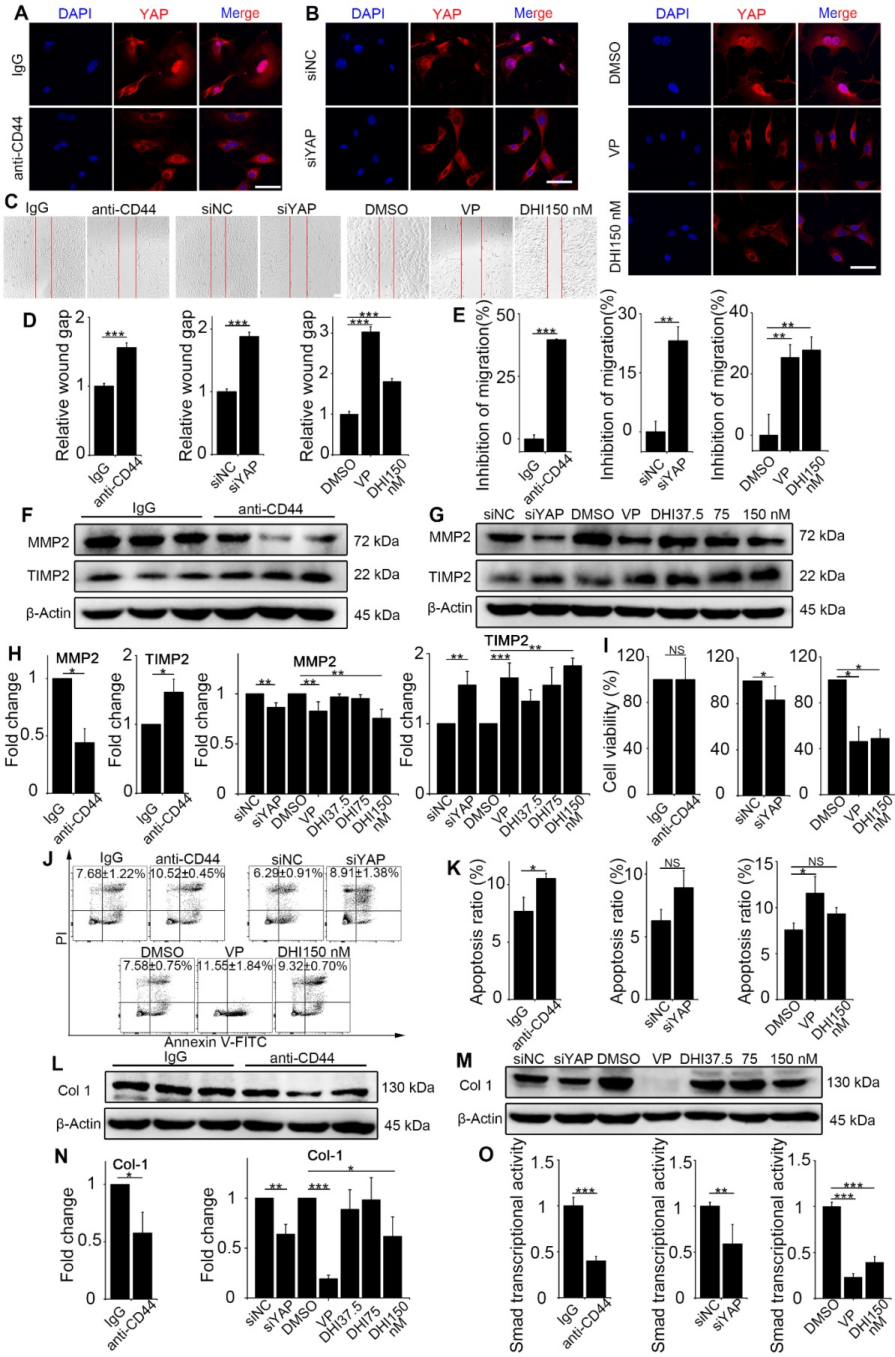
** Matrix stiffness-induced fibroblast activation is suppressed by CD44-RhoA-YAP pathway blockade.** NIH-3T3 fibroblasts were cultured on stiff (60 kappa) gel-coated coverslips treated with anti-CD44 antibody (IM7) 10 μg/mL for 12 hours, YAP siRNA for 48 hours, 250 nM VP or 37.5, 75, and 150 nM DHI for 12 hours. (**A-B**) NIH-3T3 cells were immunostained with antibody recognizing YAP. Nuclei were counter-stained with DAPI (n=3). Scale bar, 50 μm. (**C**) Wound-healing assay for determining cell migration. (**D**) Quantified wound gap of wound healing. Data shown are representative of four independent experiments. Error bars indicate mean ± SD (***, P < 0.001). (**E**) Transwell assays of cell migration inhibition. Quantified inhibition rate of migration is shown. (**F-G**) Cell lysates were subjected to immunoblotting with MMP2 and TIMP2 antibody. β-Actin was used as a loading control. (**H**) Quantification of the indicated protein level is shown. (**I**) Viability of NIH-3T3 fibroblasts was assessed by the CCK8 assay (n=5; *, P < 0.05; NS, not significant). (**J-K**) Fibroblast apoptosis was detected by flow cytometry. (**L-N**) Cell lysates were subjected to immunoblotting with the collagen-1 antibody. β-Actin was used as a loading control. (**O**) NIH-3T3 fibroblasts were transfected with a Smad4-binding element luciferase reporter; the cells were treated as described before. 48 hours post-transfection, Smad4-dependent transcription was assessed using a Smad-dependent luciferase reporter activity assay. Data shown are representative of four independent experiments. Error bars indicate mean ± SD (**, P < 0.01; ***, P < 0.001). (**E, H, K, N**) Data shown are representative of three independent experiments. Error bars indicate mean ± SD (*, P < 0.05; **, P < 0.01; ***, P < 0.001; NS, not significant).

Previous results demonstrated the upstream signaling of mechanics-induced nuclear translocation of YAP. Next, we explored whether the signaling played a vital role in matrix stiffness-induced fibroblast activation. The anti-CD44 antibody was used to block the upstream signaling, while DHI and VP were used to block nuclear translocation of YAP. Fibroblasts transfected with YAP siRNA were utilized as a positive control. DHI has been shown to inhibit nuclear translocation of YAP 17. Immunostaining analysis of the treated fibroblasts showed that regardless of upstream (Figure 4A and Figure S5A) or downstream (Figure 4B and Figure S5B) blocking, there was cytoplasmic retention of YAP.

Various functional assays of the treated fibroblasts were carried out. First, the wound-healing assay demonstrated that matrix stiffness induced a significant increase in fibroblast migration, which was inhibited by the pathway blockade (Figure 4C-D). Furthermore, the Transwell migration assay also indicated significantly increased inhibition of cell migration by the blockade (Figure 4E and Figure S5C-E). To extend these findings, we tested the protein levels of MMP2 and its inhibitor TIMP2, which play crucial roles in fibroblast proliferation, migration, and collagen deposition 18. As displayed in Figure 4F-H, there was a simultaneous decrease in the expression of MMP2 and increase in TIMP2 expression. The CCK8 assay showed no distinguishable effect on fibroblast proliferation with upstream blocking; however, downstream blocking could effectively inhibit cell proliferation (Figure 4I). Conversely, blockade of the upstream pathway rather than the downstream one could facilitate the apoptosis of NIH-3T3 fibroblasts (Figure 4J-K).

**Figure 5:**
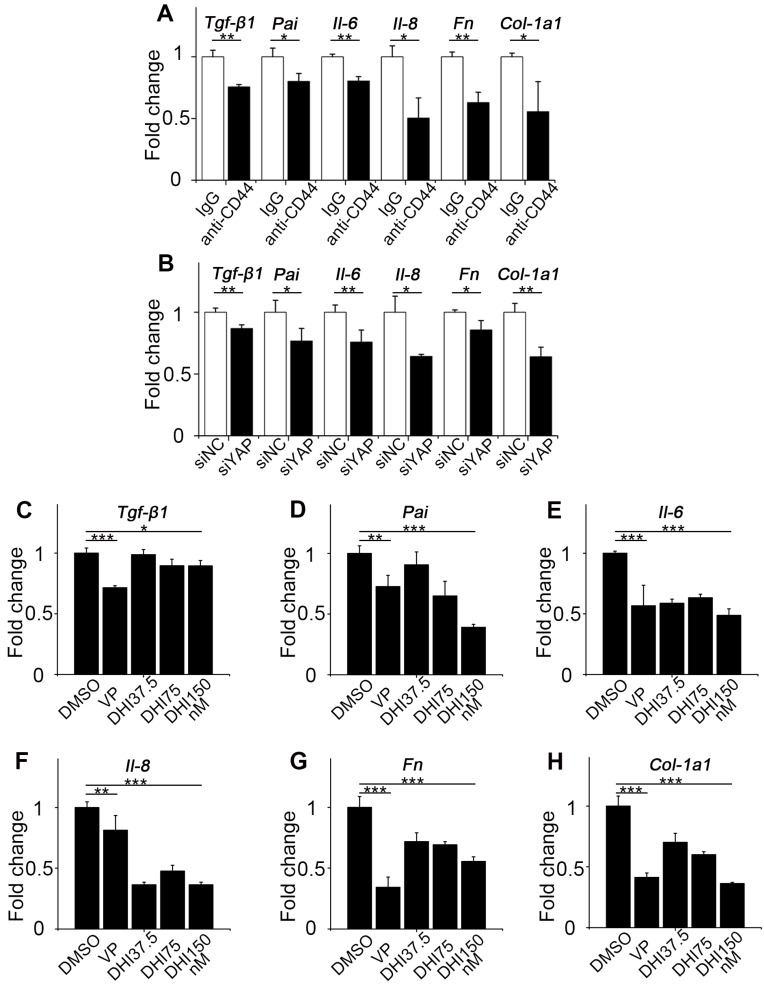
** CD44-RhoA-YAP pathway blockade reduces pro-inflammatory and pro-fibrogenic cytokines production in fibroblasts. (A**) NIH-3T3 fibroblasts grown on stiff (60 kappa) gel-coated plastic dishes were treated with anti-CD44 antibody (IM7) 10μg/mL or isotype-matched control IgG antibody for 12 hours, and qPCR analysis of *Tgf-β1, Pai, Il-6, Il-8, Fn, and Col-1a1* mRNA levels was performed. (**B**) NIH-3T3 fibroblasts grown on stiff (60 kappa) gel-coated plastic dishes were transfected with NC or YAP siRNA for 48 hours, and qPCR analysis of *Tgf-β1, Pai, Il-6, Il-8, Fn, and Col-1a1* mRNA levels was performed. (**C-H**) NIH-3T3 fibroblasts grown on stiff (60 kappa) gel-coated plastic dishes were treated with DMSO, 250 nM VP, or 37.5, 75, and 150 nM DHI for 12 hours, and qPCR analysis of *Tgf-β1* (C), *Pai* (D), *Il-6* (E), *Il-8* (F), *Fn* (G), and *Col-1a1* (H) mRNA levels was performed. (**A-H**) Data shown are representative of three independent experiments. Error bars indicate mean ± SD (*, P < 0.05; **, P < 0.01; ***, P < 0.001).

Since fibroblast activation is a major fibrogenic response to the progression of fibrosis, we measured the protein level of type I collagen, an indicator of fibroblast activation and fibrotic matrix synthesis, and found that it was decreased by pathway blockade (Figure 4L-N). It is reported that the Hippo pathway can be integrated with TGF-β-Smad signaling which is the central mediator of the fibrotic response 19. Nuclear YAP can form a complex with Smads to bind TEADs, which mediates pluripotency 20. We, therefore, tested the transcriptional activity of Smads by luciferase reporter assay. Results showed that Smads transcriptional activity was suppressed by the blockade (Figure 4O). Besides, qPCR analysis of the pro-inflammatory and pro-fibrogenic genes, such as *Tgf-β1, Pai, Il-6, Il-8, Fibronectin, and Collagen-1a1*, showed that their mRNA levels were also downregulated by upstream and downstream blocking (Figure 5). Taken together, these results confirm that the CD44-RhoA- YAP signaling pathway plays a vital role in mechanics-induced fibroblast activation by affecting migration, invasion, proliferation, apoptosis and cytokine production.

### CD44-RhoA-YAP signaling pathway blockade suppresses CS-induced silicosis

**Figure 6:**
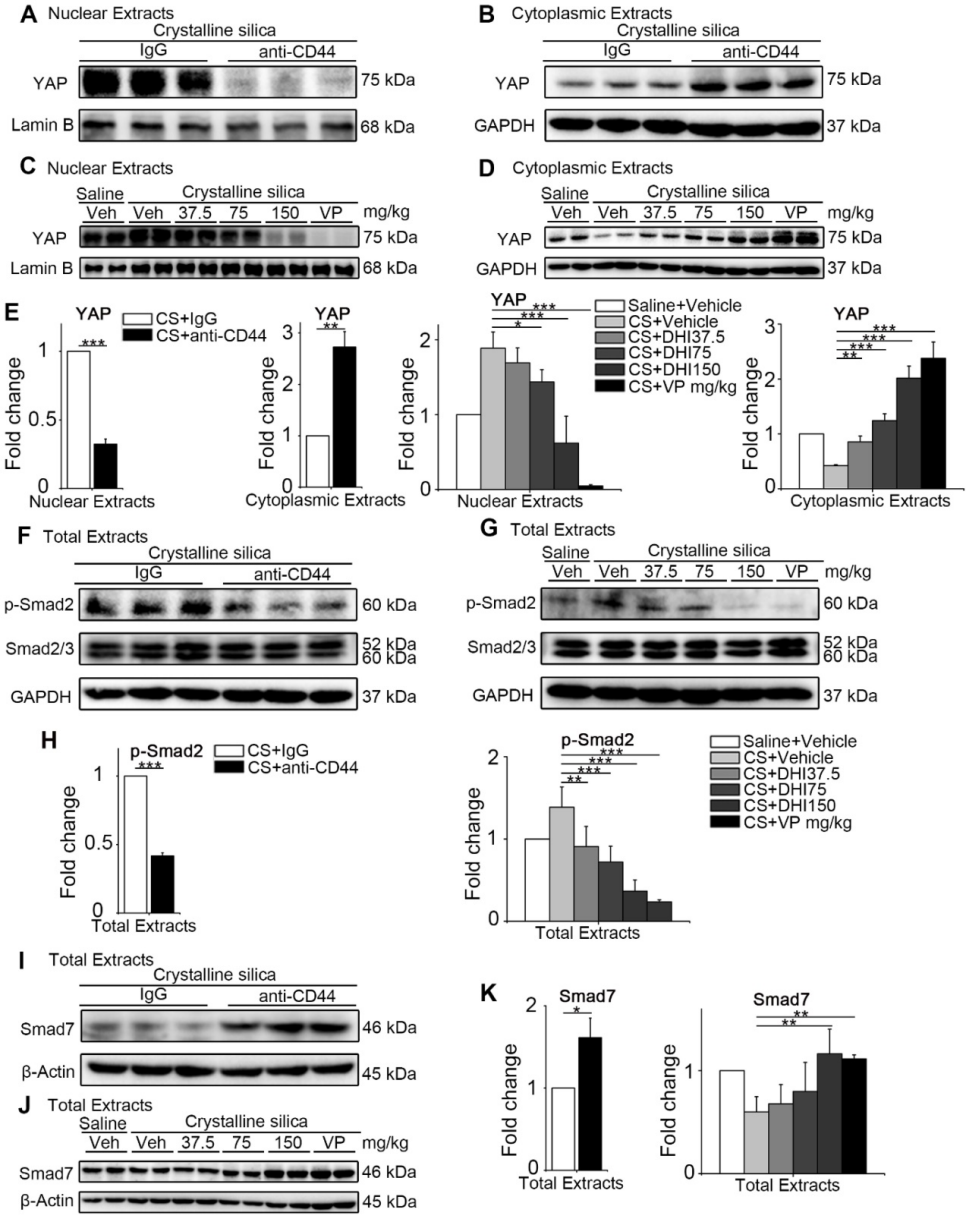
** The upstream and downstream blocking treatment relieves YAP nuclei translocation and Smad signaling activation in the lung. (A-D**) Western blot analysis of nuclear extracts (A, C) and cytoplasmic extracts (B, D) from whole lung lysates at day 56 following different treatments of mice. Analysis of YAP expression. (**E**) Quantification of YAP level is shown. (**F-G**) Western blot analysis of total extracts from whole lung lysates at day 56 following different treatments of mice. Analysis of p-Smad2 and Smad2/3 expression. (**H**) Quantification of the p-Smad2 level is shown. (**I-J**) Western blot analysis of total extracts from whole lung lysates at day 56 following different treatments of mice. Analysis of Smad7 expression. (**K**) Quantification of Smad7 level is shown. (**E, H, K**) Data shown are representative of three independent experiments. Error bars indicate mean ± SD (*, P < 0.05; **, P < 0.01; ***, P < 0.001).

Since the CD44-RhoA-YAP signaling pathway blockade could effectively protect matrix stiffness-triggered fibroblast activation* in vitro*, we examined whether it could provide a potential therapeutic benefit for postponing the development of silicosis in mice. To this end, we treated silicotic mice with anti-CD44 antibody or gradient-dose DHI as well as VP. We performed Western blot analysis of the nuclear and cytoplasmic extracts from whole lung tissues at day 14, 28 (Figure S6) and 56 to examine whether YAP was affected by the blockade. We found that the nuclear protein level of YAP was decreased while the cytoplasmic extract was increased after the blockade (Figure 6A-E). Thus, DHI could lead to decreased YAP nuclear/cytoplasmic ratio, which was similar to the effect of VP. The decrease in the ratio of nuclear/cytoplasmic YAP may indicate its inactivation.

We next determined Smad signaling activity in the lung tissues after the blockade by monitoring Smad protein levels in total lung extracts at various times. We examined p-Smad2 and found that the pathway blockade could prevent Smad2 phosphorylation without affecting Smad2/3 expression (Figure 6F-H and Figure S7A-D). The expression of Smad7, the inhibitory Smad protein, was upregulated (Figure 6I-K and Figure S7E-H) showing that the blockade treatment could prevent YAP from nuclear translocation and Smads signaling activation.

**Figure 7:**
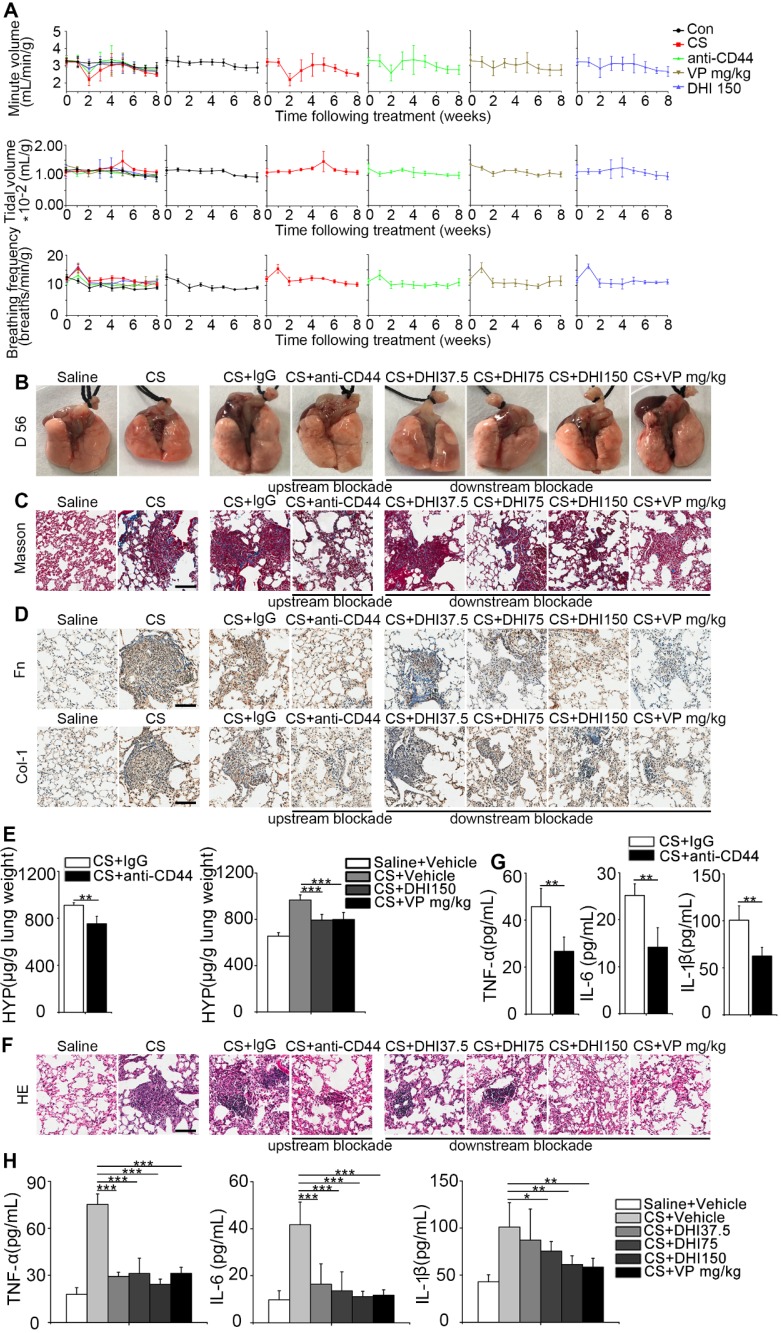
** CD44-RhoA-YAP pathway blockade improves pulmonary function and alleviates CS-induced lung inflammation and fibrosis in mice. (A**) Pulmonary function assay: minute volume, tidal volume, and breathing frequency relative to body weight in lung tissues following different treatments were measured at week 0, 1, 2, 3, 4, 5, 6, 7, and 8 (n=6 per group). (**B**) Lung morphology of upstream/downstream blockade-treated mice. (**C**) Lung sections of upstream/downstream blockade-treated mice were stained at day 56 using Masson's trichrome method (n=3 per group). Representative images of the staining are shown. Scale bar, 100 μm. (**D**) Distribution of fibronectin or collagen-1 in lung sections at day 56 after upstream/downstream blockade treatment was determined by immunohistochemical staining. Representative images of the staining are shown (n=3 per group). Scale bars, 100 μm. (**E**) Lungs of mice following different treatments were analyzed for hydroxyproline content at day 56. (n=3 per group). Error bars indicate mean ± SD (**, P < 0.01; ***, P < 0.001). (**F**) Lung sections of upstream/downstream blockade treated mice were stained at day 56 using H&E staining method (n=3 per group). Representative images of the staining are shown. Scale bar, 100 μm. (**G-H**) ELISA analysis of TNF-α, IL-6, and IL-1β in BALF (n=5 per group). Error bar indicates mean ± SD (*, P < 0.05; **, P < 0.01; ***, P < 0.001).

To evaluate the therapeutic benefit of the blockade, we used pulmonary function, which was described by minute volume, tidal volume, and breathing frequency relative to the body weight. Remarkably, the results showed that the blocking treatment led to faster recovery of the impaired pulmonary function in mice observed after CS instillation as compared to the untreated mice (Figure 7A and Table 1). Also, as shown in Figure 7B, CS treatment resulted in an apparent granular lung surface, which was significantly attenuated by the blocking process. Furthermore, Masson's trichrome staining indicated that pulmonary collagen deposition was significantly decreased (Figure 7C) which was further supported by immunohistochemical analysis of fibronectin and type 1 collagen expressions (Figure 7D). HYP measurement was also consistent with Masson's trichrome staining (Figure 7E and Figure S8A-B). Furthermore, H&E staining showed that inflammatory infiltrate was dramatically suppressed (Figure 7F) which was supported by ELISA analysis of the pro-inflammation cytokines TNF-α, IL-6, and IL-1β expression in BALF (Figure 7G-H and Figure S8C-D). These observations demonstrate that the blockade not only affects CS-induced pulmonary fibrogenesis but also alleviates chronic pulmonary inflammation *in vivo*.

## Discussion

**Figure 8:**
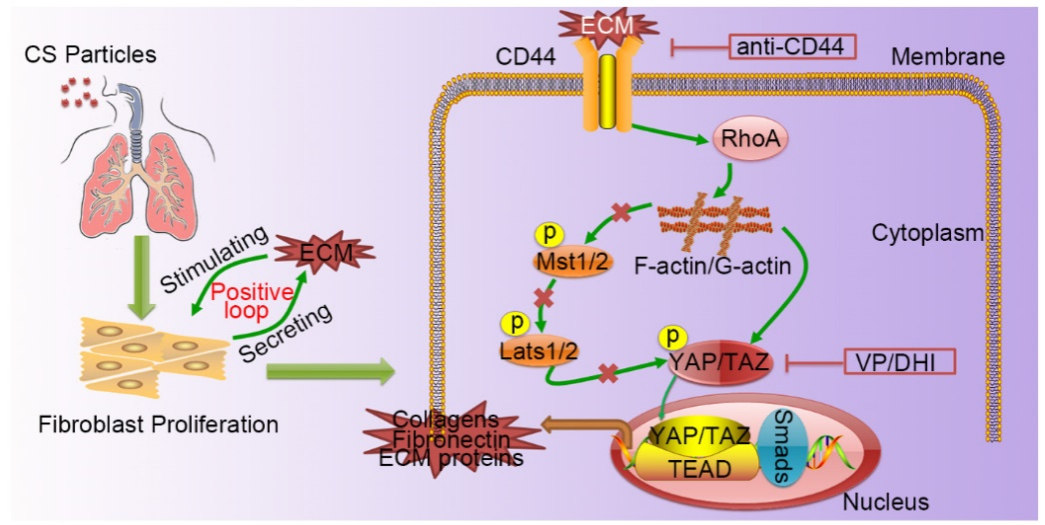
** A schematic model for the positive feedback loop between fibroblast activation and ECM deposition mediated by CD44-RhoA-YAP signaling pathway.** Activated fibroblasts through CD44-RhoA-YAP signaling pathway drive ECM production and accumulation further stimulating fibroblast activation. Upstream blockade by anti-CD44 antibody or downstream blockade by DHI or VP delays CS-induced lung fibrosis.

Silicosis is a non-reversible process ranging from chronic inflammation to progressive fibrosis that ultimately leads to lung destruction caused by excessive ECM deposition 21. Although silicosis maintains a significant inflammatory response throughout the course of the disease, a growing body of evidence suggests that once fibrosis has established, anti-inflammation therapy alone is not enough to postpone fibrosis 2, 22, 23. Furthermore, isolated anti-inflammatory therapy may worsen clinical outcomes in patients with lung fibrosis 24. Recent research demonstrated that excessive ECM- forming mechanical cues play an essential role in promoting fibroblast activation and perpetuating fibrotic pathologies 25-27. Hence, therapeutically targeting tissue mechanics, either by directly altering the mechanical cues presented to cells or by disrupting the cellular signaling to mechanics will provide a potential therapeutic strategy. Although it has previously been reported that mechano-signaling through YAP drives fibroblast activation, the upstream and downstream components of mechano- activation pathway remained to be elucidated. In this study, we demonstrated CD44-RhoA-YAP signaling could facilitate ECM-induced fibroblast activation (Figure 8) and blocking this signaling pathway could ameliorate CS-induced experimental silicosis *in vivo*.

YAP is the prime mediator of the Hippo pathway and its expression and cellular distribution were reported to be mechanically regulated by stiffness of the extracellular matrix 28-30. We demonstrated that YAP was upregulated and nuclear- localized in lungs of CS-injured mice, especially in spindle-shaped cells (fibroblasts), during fibrosis when mechanical forces were high (Figure 1). As an intracellular molecule, YAP is not a direct receptor for surrounding mechanical forces. Integrin receptors have been identified as cell surface receptors that regulate the Hippo-YAP pathway. As the ECM can activate integrin-mediated downstream signaling, targeting integrin is logically the first choice to interrupt ECM mechano-sensing, since these transmembrane adhesion proteins can directly connect to the extracellular matrix and transduce bidirectional signaling. However, a previous study indicated that integrin expression in fibroblasts does not alone drive the cellular response to increased mechanics 31. Besides, selecting which integrins to target to block matrix stiffness sensing is not straightforward, since there are diverse arrays of integrin heterodimers 32. Thus, identifying another sensor to blunt surrounding mechanical forces appears extremely significant.

In this study, we showed, for the first time, that CD44, a transmembrane adhesion receptor involved in the regulation of YAP, was upregulated in fibroblasts response to mechanical forces and matrix stiffness. CD44 is recognized as a receptor for hyaluronic acid (HA) 33, 34, and can also bind to a variety of other ECM components, including type I and type III collagens 35, fibronectin 36, and laminin 37. It is of note that a previous report indicated co-localization of CD44 and β1 integrins in the cell membrane 36 indicating they may function synergistically and affect fibroblast activation. Furthermore, the involvement of CD44 in an invasive phenotype of fibroblasts and promoting the progression of fibrosis has also been described 38 which is consistent with our results. We believe that our results may provide a deeper molecular understanding of the mechanisms underlying the phenotype switching.

The bioinformatic analysis provided clues for our present study that CD44 might act through Rho GTPases to regulate YAP activity. Rho GTPases, including RhoA, Rac1, and Cdc42, are critical regulators of cytoskeletal dynamics and cellular force through the formation of actin stress fibers. We and others found that CD44 could activate RhoA, and, to a lesser extent, Rac1 and Cdc42 signaling 39, 40. However, other reports suggested that CD44 could affect all three Rho GTPases 41, 42. To resolve this issue, we performed siRNA-mediated knockdown of RhoA, Rac1, and Cdc42. Remarkably, only RhoA could alter downstream signaling and RhoA activation could effectively reverse CD44 knockdown effects, which can, in turn, be reversed by F-actin inhibitor Lat A (Figure 3). Thus, CD44-RhoA-F-actin signaling may at least partially be involved in regulating YAP in mechanical stimuli-induced fibroblast activation. Canonical Hippo cascade indicated that upstream stimuli could promote phosphorylation of the kinase Mst1/2 and Lats1/2 further controlling the cytoplasmic retention and degradation of YAP/TAZ 43, 44. However, we found that CD44- RhoA modulated YAP activation directly via F-actin polymerization/ depolymerization independent of the Hippo kinase cascade, which was consistent with a previous investigation 9. To our knowledge, our study shows, for the first time, that mechanical stimuli may modulate YAP activation through CD44-RhoA- F-actin independent of canonical Hippo cascade.

Fibroblast activation is hypersensitive to multiple mediators 4, 45, among which TGF-β/Smad signaling plays a central role 46, 47. The cross talk between Hippo and TGF-β signaling has been shown to play a significant role in intracellular signaling and transcriptional regulation. TGF-β intracellular signaling proceeds through phosphorylation of Smad2 and Smad3, which interact with Smad4 and translocate to the nucleus. The nuclear Smad complexes then bind to various transcription factors regulating the transcription of profibrotic genes. In the cytoplasm, phosphorylated YAP sequesters Smad2/3 to prevent nuclear accumulation and inhibits TGF-β responsiveness, whereas, in the nucleus, YAP forms a p-Smad2/ 3-Smad4 complex and participates in fibroblast activation 19. In this context, our result of the luciferase reporter assay indicated that the blocking treatment significantly reduced Smad4 transcription in fibroblasts which resulted in the reduction of mechanics- induced fibroblast activation.

Recognizing the role of CD44-RhoA-YAP signaling in mechanics-induced fibroblast activation, we attempted to elucidate its potential therapeutic benefits by using the mouse model of silicosis. A recent study identified the stiffness-dependent role of the Rho-associated kinase (ROCK) in driving the conversion of fibroblasts to myofibroblasts during fibrosis 48. In this signaling pathway, we chose CD44 and YAP and not RhoA, as our molecular targets, since Rho is considered “undruggable” as its three-dimensional structure lacks hydrophobic clefts 49. Instead of ROCK, the major downstream effector of Rho was used as an alternative target. On this basis, we chose to treat mice with upstream blocking antibody and downstream blockers VP/DHI. We noticed that upstream blockade with anti-CD44 resulted in greater nuclear translocation of YAP compared with the downstream blockade, while the phosphorylation of Smad2 in the mice lungs was comparable between the two modalities. However, the upstream blocking with anti-CD44 manifested in more significant therapeutic effects with fewer collagen deposition and faster pulmonary function recovery. It was probably attributed to the fact that CD44 expression is not limited to fibroblasts. Other cell types, such as lymphocytes, macrophages, and endothelial cells 50, 51, also express CD44 in the lung, which can be targeted by the blocking antibody.

Interestingly, when comparing the two downstream blocking effects of DHI and VP, we found DHI exerted a lesser effect on YAP translocation, while the subsequent Smad2 signaling activity suppression, as well as the pulmonary function recovery, were almost identical between the two treatments.

It is possible DHI has multi-molecular targets that need to be further investigated. It should be noted that either treatment could reduce the pro-inflammatory cytokines in BALF. Thus, it is a disadvantage of the treatment that neither upstream nor downstream blockades directly target the fibroblasts. On the other hand, blocking multiple targets may be beneficial for better therapeutic efficacy.

In the future, specific localized therapeutics to the target organ or specific cell types for precise treatment are highly desirable to minimize the potentially deleterious side effects. Considering the chronic inflammation characteristic of silicosis, we believe that using combination therapies with anti- inflammatory and anti-mechanics directed at more than one specific target may provide a better therapeutic benefit. In summary, our study elucidates potential novel molecular mechanism to mechanics- induced fibroblast activation and proposes attractive new therapeutic strategies for intervening the progression of silicosis.

## Supplementary Material

Supplementary figures and tables.

## Figures and Tables

**Table 1:** Effects of various treatments on the pulmonary function in mice.

**Minute volume relative to body weight (mL/min/g)**					
	**Saline**	**CS**	**anti-CD44**	**VP**	**DHI 150 mg/kg**
**0**	3.30±0.15	3.23±0.18	3.31±0.17	3.26±0.33	3.22±0.18
**1**	3.23±0.31	3.21±0.22	3.28±0.34	3.19±0.31	3.21±0.25
**2**	3.14±0.12	2.20±0.35	2.58±0.46	2.82±0.42^#^	2.86±0.54^#^
**3**	3.21±0.16	2.73±0.62	3.28±0.51^*^	3.12±0.27^NS^	3.12±0.41^NS^
**4**	3.21±0.17	3.05±0.66	3.34±0.84	2.99±0.40	3.11±0.39
**5**	3.18±0.21	3.07±0.38	3.24±0.34	3.14±0.59	3.12±0.54
**6**	2.93±0.17	2.87±0.20	2.93±0.24	2.79±0.25	2.91±0.32
**7**	2.87±0.16	2.60±0.18	2.76±0.22	2.71±0.32	2.72±0.18
**8**	2.88±0.32	2.50±0.12	2.76±0.26	2.72±0.31	2.66±0.28
Vertical comparison: *, P < 0.05, week 3 compared to week 2 among anti-CD44 treated mice; NS, not significant, week 3 compared to week 2 among VP or DHI treated mice; Horizontal comparison: #, P < 0.05, VP or DHI treated mice compared to CS instillation mice after week 2.					

Tidal volume relative to body weight *10^-2^ (mL/g)					
	**Saline**	**CS**	**anti-CD44**	**VP**	**DHI 150 mg/kg**
**0**	1.17±9.77E-2	1.11±0.136	1.23±0.149	1.33±3.39E-2	1.13±0.224
**1**	1.20±5.50E-2	1.14±4.32E-2	1.04±7.96E-2	1.23±6.85E-2	1.13±9.05E-2
**2**	1.16±2.25E-2	1.13±6.83E-2	1.11±4.22E-2	1.04±8.38E-2	1.13±7.36E-2
**3**	1.15±8.26E-2	1.21±4.32E-2	1.19±5.12E-2	1.14±3.92E-2	1.21±0.309
**4**	1.15±9.11E-2	1.25±5.16E-2	1.10±0.167	1.15±7.11E-2	1.27±0.317
**5**	1.16±5.01E-2	1.47±0.337	1.07±7.14E-2^*^	1.08±0.100^*^	1.17±0.102^NS^
**6**	1.00±1.94E-2	1.20±4.41E-2	1.04±2.76E-2	0.99±4.83E-2	1.10±0.106
**7**	0.98±5.08E-2	1.14±9.00E-2	1.00±4.63E-1	1.06±0.106	1.01±0.132
**8**	0.94±0.152	1.13±0.224	1.00±0.101	1.02±8.67E-2	0.98±0.124
Horizontal comparison: *, P < 0.05, anti-CD44 or VP treated mice compared to CS instillation mice after week 5; NS, not significant, DHI treated mice compared to CS instillation mice after week 5.					

**Breathing frequency relative to body weight (breaths/min/g)**					
	**Saline**	**CS**	**anti-CD44**	**VP**	**DHI 150 mg/kg**
**0**	12.80±0.87	12.13±0.79	11.18±0.86	11.97±1.20	11.91±0.98
**1**	11.39±0.75	15.48±1.42	13.27±1.57	15.75±1.60	16.29±0.80
**2**	9.11±1.19	11.34±1.37^***^	10.01±1.12_NS_^**^	10.79±1.50_NS_^***^	10.74±1.37_NS_^***^
**3**	10.18±0.62	11.75±0.73	10.42±1.03	10.62±1.32	10.60±1.28
**4**	8.95±0.87	12.39±0.77	10.01±1.08	10.72±1.24	10.46±1.67
**5**	9.38±0.42	12.28±0.34	9.71±0.68	10.12±1.55	11.54±1.16
**6**	8.55±0.39	11.28±0.55	10.21±0.77	9.62±0.76	11.08±0.61
**7**	8.79±0.38	10.56±0.76	9.63±0.79	11.21±1.62	10.96±0.42
**8**	9.14±0.50	10.27±0.70	10.95±1.21	11.44±1.57	11.19±0.79
Vertical comparison: ***, P < 0.001; **, P < 0.01, week 2 compared to week 1 among CS, anti-CD44, VP, or DHI treated mice; Horizontal comparison: NS, not significant, anti-CD44, VP, or DHI treated mice compared to CS instillation mice after week 2.					

## Abbreviations

Cdc42: cell division control protein 42 homolog
F-actin: filamentous actin
Il-6: interleukin 6
Il-8: interleukin 8
Lat A: latrunculin A
Lats: large tumor suppressor
MMP2: matrix metalloprotein 2
Mst: mammalian STE20-like protein kinase
Pai: plasminogen activator inhibitor
Rac1: ras-related C3 botulinum toxin substrate 1
RhoA: ras homolog gene family member A
siNC: negative control siRNA
siRNA: small interfering RNA
TAZ: PDZ-binding motif
TEAD: TEA domain transcription factor
Tgf-β: transforming growth factor beta
TIMP2: tissue inhibitor of metalloproteinase 2
