# Supplementary material for: Targeting Mechanics-Induced Fibroblast Activation through CD44-RhoA-YAP Pathway Ameliorates Crystalline Silica-Induced Silicosis
Source: Theranostics. 2019 Jul 9;9(17):4993–5008. doi: 10.7150/thno.35665 (PMC6691376; doi:10.7150/thno.35665)
Supplement: Supplementary file 1 — Supplementary figures and tables. [file thnov09p4993s1.pdf]

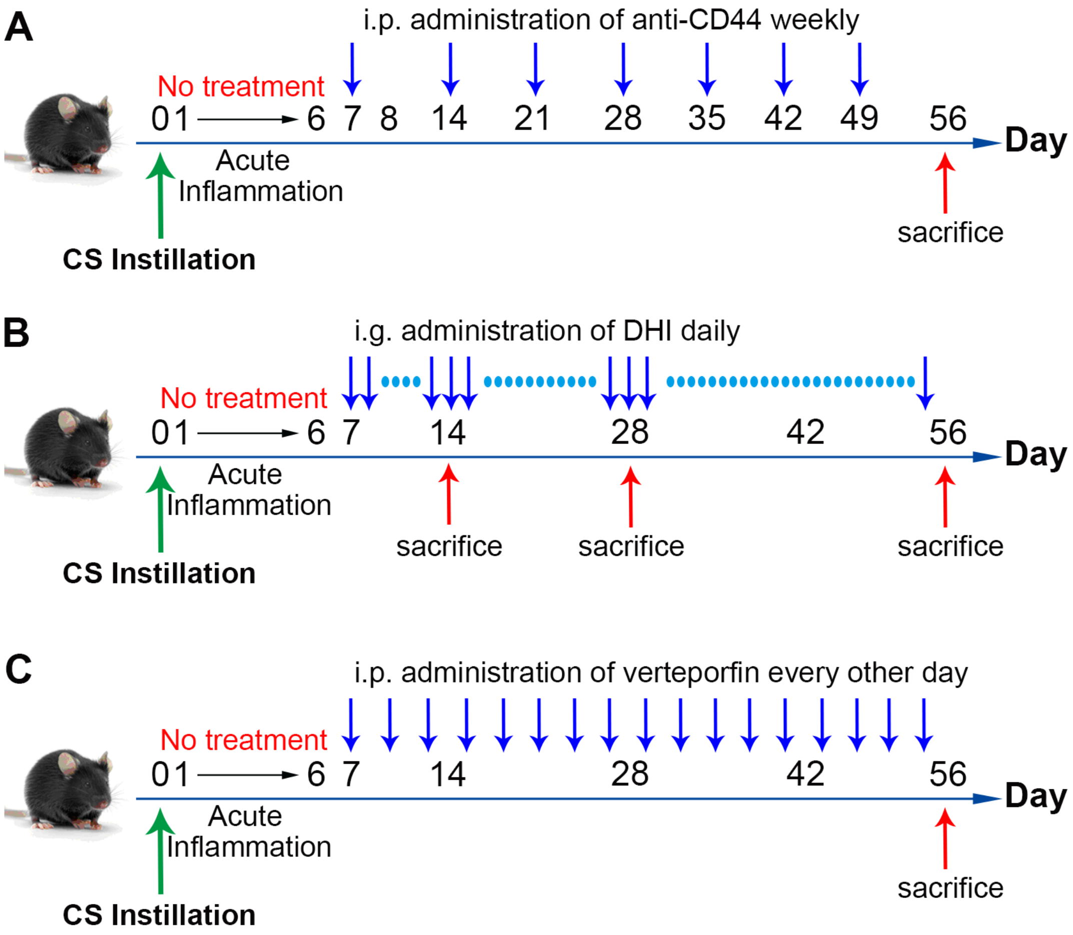

Supplementary figure 1. (**A**) Male C57BL/6 mice received weekly intrapertoneal imjection of anti-CD44 or isotype-matched control antibody (300 μg in 500 μl saline). (**B**) Male C57BL/6 mice received daily intragastrical administration of gradient-dose DHI or vehicle. (**C**) Male C57BL/6 mice received intrapertoneal imjection of verteporfin or vehicle every other day. (**A-C**) All treatments were beginning at day 7 after CS instillation (n=10 per group).

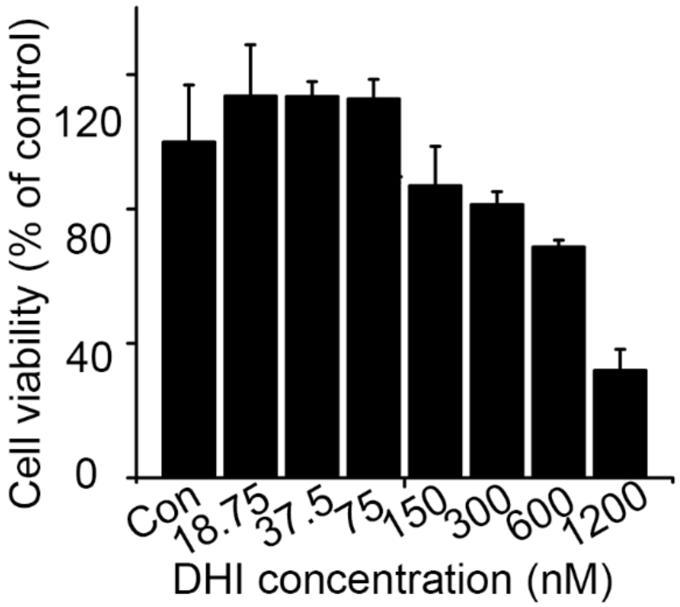

Supplementary figure 2. Viability of NIH-3T3 fibroblasts incubated with different concentrations of DHI was detected by the MTT assay (n=3; *, $P < 0.05$).

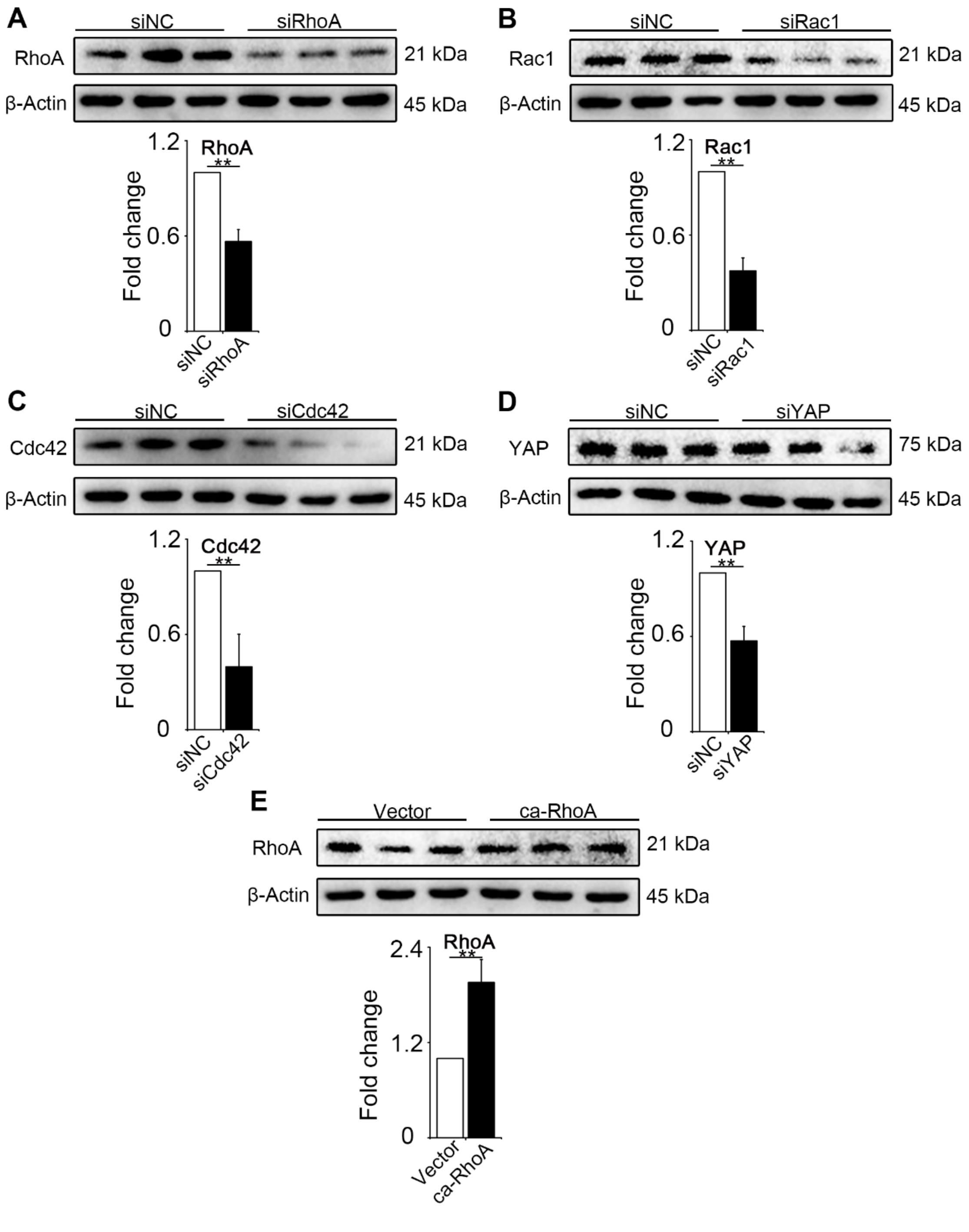

Supplementary figure 3. (**A-E**) Western blot analysis of RhoA (A), Rac1 (B), Cdc42 (C), YAP (D) siRNA and ca-RhoA (E) plasmid transfection efficiency. β-Actin was used as a loading control. Data shown are representative of three independent experiments. Error bars indicate mean ± SD (**, $P < 0.01$).

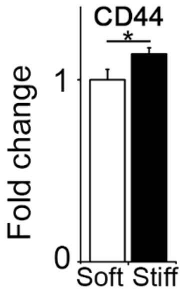

Supplementary figure 4. NIH-3T3 fibroblasts were cultured on soft (1 kappa) or stiff (60 kappa) gel-coated coverslips and qPCR analysis of *Cd44* mRNA level was performed. Data shown are representative of three independent experiments. Error bars indicate mean ± SD (*, $P < 0.05$).

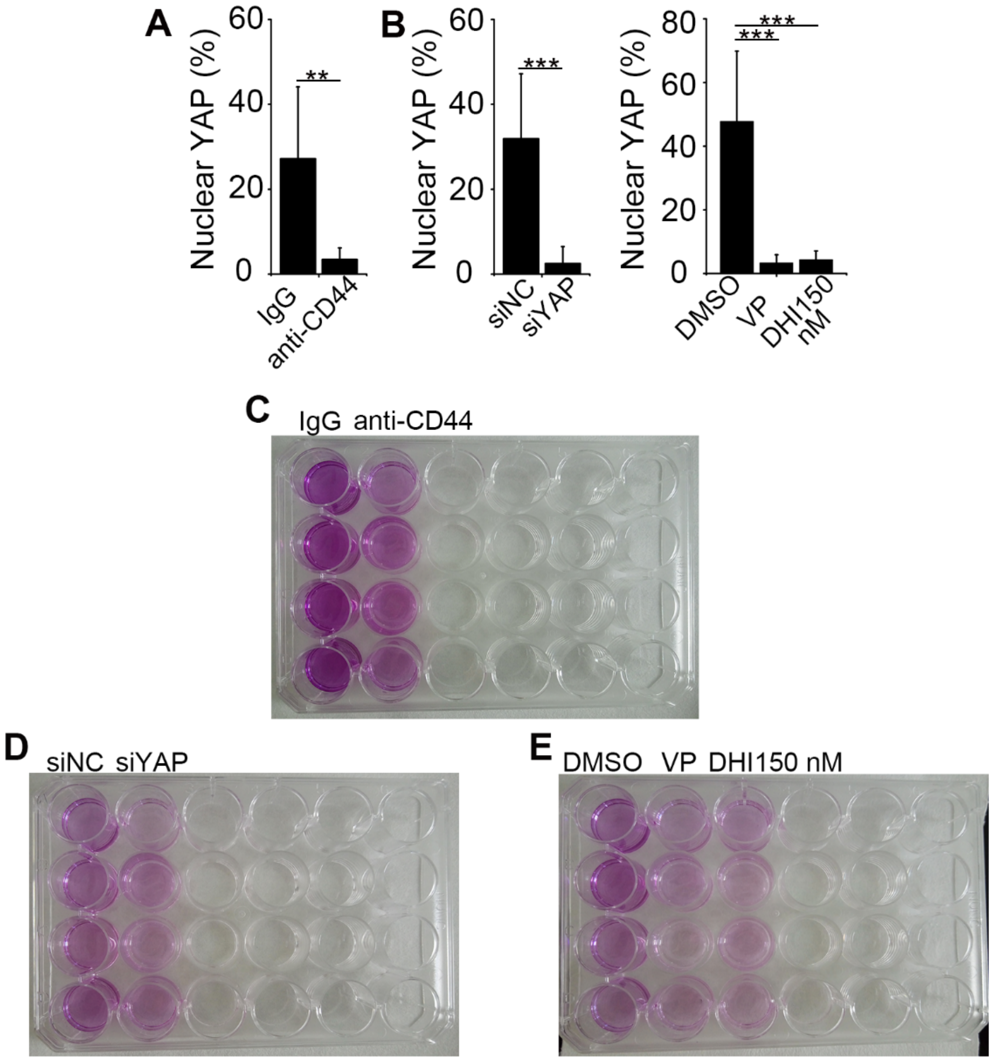

Supplementary figure 5. NIH-3T3 fibroblasts were cultured on stiff (60 kappa) gel-coated coverslips treated with anti-CD44 antibody (IM7) 10 μg/mL for 12 hours, YAP siRNA for 48 hours, 250 nM VP or 150 nM DHI for 12 hours. (**A-B**) NIH-3T3 cells were immunostained with an antibody recognizing YAP. The percentage of cells with predominantly nuclear YAP staining was quantified (n=3; **, $P < 0.01$; ***, $P < 0.001$). (**C-E**) Example images about Transwell migration assay in the different treated fibroblasts as described in figure 4E were shown.

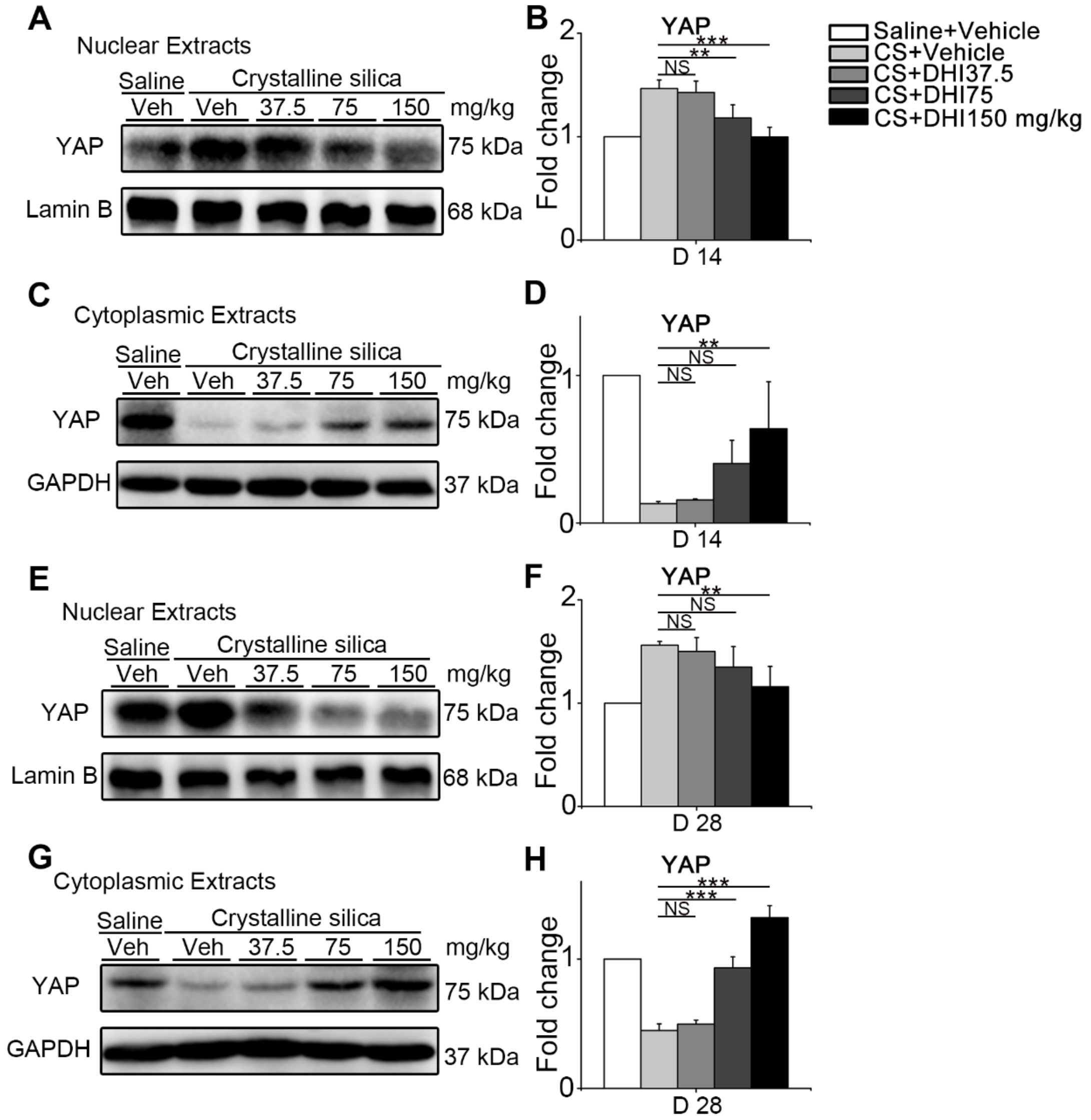

Supplementary figure 6. (**A-D**) Western blot analysis of nuclear extracts (A-B) and cytoplasmic extracts (C-D) from whole lung lysates at day 14 following gradient-dose DHI treatments of mice. Analysis of YAP expression. (**E-H**) Western blot analysis of nuclear extracts (E-F) and cytoplasmic extracts (G-H) from whole lung lysates at day 28 following gradient-dose DHI treatments of mice. Analysis of YAP expression. (**B, D, F, H**) Data shown are representative of three independent experiments. Error bars indicate mean ± SD (**, $P < 0.01$; ***, $P < 0.001$; NS, not significant).

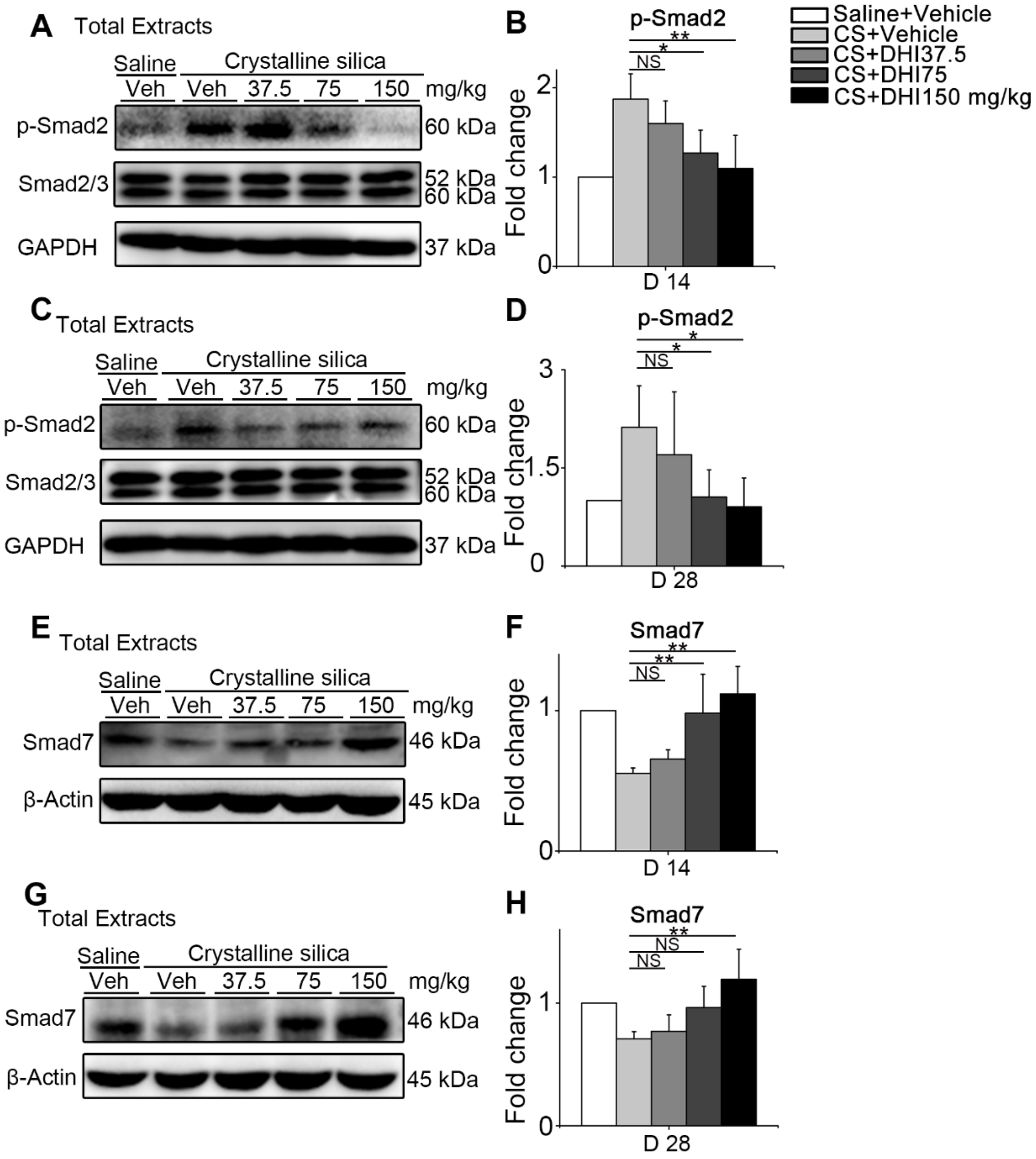

Supplementary figure 7. (**A-D**) Western blot analysis of total extracts from whole lung lysates at day 14 (A-B) or 28 (C-D) following gradient-dose DHI treatments of mice. Analysis of p-Smad2 and Smad2/3 expressions. (**E-H**) Western blot analysis of total extracts from whole lung lysates at day 14 (E-F) or 28 (G-H) following gradient-dose DHI treatments of mice. Analysis of Smad7 expression. (**B, D, F, H**) Data shown are representative of three independent experiments. Error bars indicate mean ± SD (*, $P < 0.05$; **, $P < 0.01$; NS, not significant).

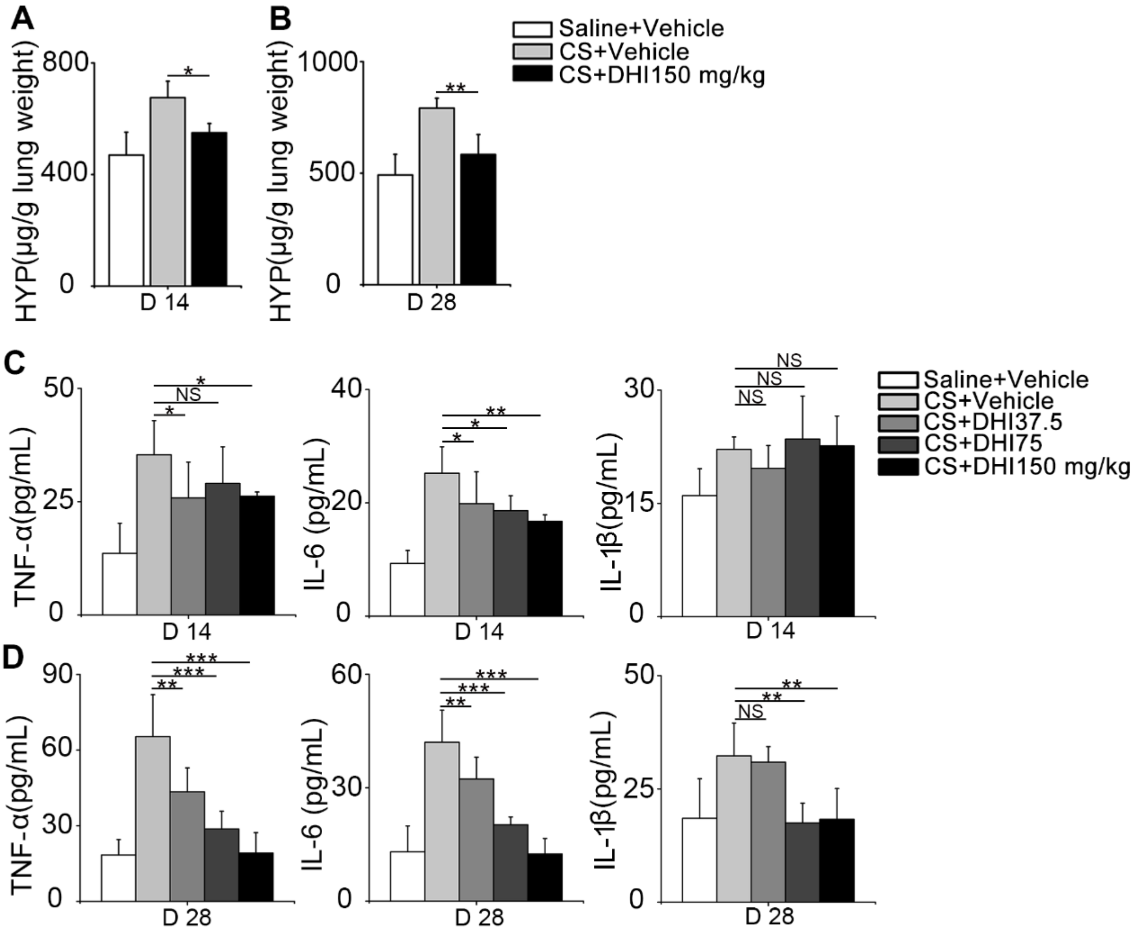

Supplementary figure 8. (**A-B**) Lungs of different treatments of mice were analyzed for hydroxyproline content at day 14 (A) and day 28 (B) (n=3 per group). Error bars indicate mean ± SD (*, P < 0.05; **, P < 0.01). (**C-D**) ELISA analysis of TNF-α, IL-6 and IL-1β in BALF at day 14 (C) and day 28 (D) following gradient-dose DHI treatments of mice (n=5 per group). Error bars indicate mean ± SD (*, P < 0.05; **, P < 0.01; ***, P < 0.001; NS, not significant).

Supplementary table 1. Primary antibodies used for western blot, immunofluorescence and immunohistochemistry.

Antibody	Company	Catalog #	Application	Dilution
YAP	Cell Signaling Technology	#14074	WB	1:1000
RhoA	Cell Signaling Technology	#2117	WB	1:1000
Rac	Cell Signaling Technology	#2465	WB	1:1000
Cdc42	Cell Signaling Technology	#2466	WB	1:1000
Mst 1	Cell Signaling Technology	#3682	WB	1:1000
Lats 1	Cell Signaling Technology	#3477	WB	1:1000
Phospho-Smad2	Cell Signaling Technology	#3108	WB	1:1000
Smad2/3	Cell Signaling Technology	#8685	WB	1:1000
Lamin B1	Cell Signaling Technology	#13435	WB	1:1000
GAPDH	Cell Signaling Technology	#2118	WB	1:1000
β-Actin	Cell Signaling Technology	#4970	WB	1:1000
MMP2	R&D systems	AF1488	WB	0.1 ug/mL
TIMP2	R&D systems	AF971	WB	1 ug/mL
Smad7	R&D systems	MAB2029	WB	1 ug/mL
Collagen 1	Absin Bioscience	abs131984	WB	1:500
CD44	Abcam	ab119348	IF	1:100
YAP	Santa Cruz	sc-101199	IF	1:50
α-SMA	Abcam	ab32575	IF	1:1000
YAP	Cell Signaling Technology	#14074	IHC	1:100
Collagen 1	Absin Bioscience	abs131984	IHC	1:100
Fibronectin	Novusbio	NBP1-91258ss	IHC	1:200

WB: western blot; IF: immunofluorescence; IHC: immunohistochemistry;

Supplementary table 2. Quantitative PCR primers for analysis.

Gene	Primer forward	Primer reverse
Gapdh	AGGTCGGTGTGAACGGATTTG	TGTAGACCATGTAGTTGAGGTCA
Tgf-β1	CTCCCGTGGCTTCTAGTGC	GCCTTAGTTTGGACAGGATCTG
Pai	ACGGTGATGCGATATAATGTAAACG	CATTCCTGAGAAACACAGCATTG
Il-6	CAACGATGATGCACTTGCAGA	CTCCAGGTAGCTATGGTACTCCAGA
Il-8	TTCTTGTCTTTCAGCATGGC	GAACGTGACCTCTTTCTCCC
Fibronectin	GCAGTGACCACCATTCCTG	GGTAGCCAGTGAGCTGAACAC
Collagen-1a1	GCTCCTCTTAGGGGCCACT	CCACGTCTCACCATTGGGG
